# East and West

**Published:** 1887-12-10

**Authors:** Leslie Keith

**Affiliations:** Author of "The Chilcotes," "Uncle Bob's Niece," etc.


### EAST AND WEST.

BY LESLIE KEITH.

Author of "The Chilcotes," "Uncle Bob's Niece," etc.

(*Continued from p.* 172.)

CHAPTER.—XI.—THE BEGINNING OF THE END.

UNDOUBTEDLY even Felix Mervyn, who ought of course to have been wholly given over to the study of germs, felt the charm of shared confidences stirring in his blood, and walked briskly about as if he were the possessor of a private joy. A new current had set into his life, and half-unconsciously his thoughts and hopes were shaping themselves in a new direction. He had a habit of reading far into the night, taking what he could of the science of his art along with its practice, which he rightly valued as the more important; and hitherto somebody's treatise on fevers, and somebody else's deliverances on rheumatic gout had been all-absorbing. How was it, then, that a young lady in grey, with sombre grey eyes, so frequently presumed to cross the page, and to linger there too persistently for the student's peace?

Under these conditions the day was held blank that did not record a meeting; there was undoubtedly—as there always is—a good deal of illness in Baron Street and the alleys that led from it, and that may have accounted for the young man's frequent presence there. He lived within a stone's throw of the tenement where David and Mary had drifted, in an old, old home that, by reason of its being built as a vicarage, had maintained its respectability. Mervyn shared it with the priest of curious vestments and narrow views—occupying a comfortable set of rooms, and refusing to practise with the latter a mortifying self-denial in the matter of food and furniture.

"I draw the line at copying the manners and habits of my patients," he said. "To make myself a pig will scarcely help them."

"You can reach them better if you place yourself on their level," said the priest, who was lean as an Aquinas by reason of a studiously sparing diet.

"I don't see it," said Mervyn sturdily. "I prefer lifting them up to my level: if I were to reduce myself to theirs I should presently break down, and what good would that do to the poor beggars? They'd rather I made them well than fall ill myself out of sympathy."

The doctor was guilty of grinning over the glass of wine which he could not persuade the priest to share. "I shall have you on my hands presently," he thought to himself; and on the whole it seemed to him that there was more wisdom in his snug cellar of good wine, his easy chair, his writing-table by Gillow, his well-picked library, and even his "pickled vermin," than in the young clergyman's severely practical rigours. And his dreams of Alicia, and his meetings with her—was there wisdom or folly in these?

Alicia, too, had come to count upon those interviews, that were not, perhaps, left wholly to chance. In her new dependence she had learned to lean upon his superior experience; he was an encyclopædia to be dipped into on all occasions—an answerer of riddles; if as yet he was nothing more, that was because her thoughts had so forcibly taken a new channel that she had no room in them for love.

Her unconsciousness saved her from that embarrassing shyness that is usually the beginning of a mutual attraction. She could look at Felix directly, and meet him without so much as an inward tremor or an outward blush.

She was lingering purposely one day at the ever-open door, hoping to intercept him, when he appeared coming out of a narrow lane, walking with that brisk and confident step that was so different from the shuffling footfall of the native of Bethnal Green. There was a smile of whimsical humour on his face, which deepened into pleasure at sight of her.

"I've been settling a family feud," he said as he shook hands with her; "that is part of a doctor's duties here. Mrs. Jones, in a moment of excitement, flung the teapot at her lord, and cut open his head."

"That was a forcible argument," said Alicia, with a relaxing of the grave mouth. "How did you settle the matter?"

"Patched up Jones's head, and told him he ought to preserve family discipline."

"Therein you showed your bachelor ignorance," said Alicia, with a shake of the head. "If you had inquired a little further you would have very likely found that Mrs. Jones was quite right."

"That sort of ignorance may be amended," said Mervyn, putting a little more earnestness into his voice; but Alicia had an anxiety of her own that occupied her.

"I want you to go, please, and see David Jardine," she said. "Mary is out and he is alone. Could you find some excuse for paying him a visit?"

"I will ask him to read one of his poems."

"Very well," said Alicia, not allowing herself to smile. "I will wait here till you come back."

He obeyed her at once, running lightly up the dark stairs and making his appeal to the poet, whom it scarcely seemed to surprise.

Alicia was waiting on the same spot when he returned.

"I am very sorry to have kept you," he said, "but I couldn't get away under two poems."

She turned inquiringly towards him, and he said in answer to the unspoken question:

"Couldn't you hurry up Short and Long with that book of his?"

A startled wonder crept into the girl's eyes.

"Do you mean ——" she began.

"It has been a trying summer—he should never have come here. The changed life and the disappointments, and one thing and another, have hurried on matters. That is all. The mischief was done before he came here; very likely even if he had remained in Scotland he could not have faced another winter."

"What shall I do?" asked Alicia, with an appealing submission that was very winning.

"There is nothing to be done, I'm afraid."

"Will he—leave us soon?" she asked fearfully.

"I think he will," he answered gravely.

"But he can't die there, in that hot, close little room; that would be too sad. And Mary will not let me do anything."

Mervyn checked a smile. In Mary's independence he recognized a pride that ran in the family blood.

"The people who can live here do not find it so hard to die here," he said. "They have no choice, as a rule, unless it is their good fortune to get into the hospital."

"David would not go into a hospital."

"Even if he were willing, he isn't strictly a hospital case."

"Isn't every case of sickness among the poor and destitute a hospital case? That is what we are told when we are urged to give our half-crowns."

"It is so ideally; it might be so in practice if your half-crowns were more numerous. Hospitals subsist mainly on public generosity: as the matter stands, there is scarcely one in London that isn't deep in debt, nor one that hasn't a struggle for existence. Have you never been told these things from your pulpit?"

"I tell you," she said, "we don't listen to sermons. Our clergy are too politic to offend our ears with rude details, and if here and there one is bold enough to tell us the truth, and move us by his earnestness on Sunday, the impression is clean gone by Monday. It doesn't do for us to have long memories. If we remembered these things we couldn't live in society."

"Then society has a conscience, after all?"

But she was not listening. "Do you mean to tell me that here—in rich London—the hospitals are starved?"

"I mean it. If you will go with me to-morrow to St. Placide's I will show you whole wards shut up and useless—not for lack of patients, but because the half-crowns that might have kept them open have failed."

"Oh," said Alicia, "what a wicked world it is!"

"No," he corrected her; "it is a thoughtless, heedless world if you will, but it has a sound heart at bottom. There never was a time in its history when people gave more, or were more spontaneously benevolent. The worst of it is, their gifts are filtered into so many different channels that a hundred good causes get a dribble, and not one a satisfactory draught."

All this did not appear to mend David Jardine's case. Death is naturally a very dreadful mystery to the young, and it had the awfulness of the unfamiliar to Alicia as it necessarily no longer had for the doctor. To yield up life in the stifling London lodging, with the sights and sounds of a hopeless conflict seen and heard to the last, seemed to her too sad a fate to be tolerated for her cousin, and after some pondering she decided upon a plan of action.

In the first place, however, she left Bethnal Green at a much earlier hour than usual. Mary's heart sank as she saw her go—it was natural enough she should read in this the first signs of flagging in Alicia's interest. Well, who could be fool enough to dream that it would last—that one so brilliant and prosperous, so fitted to shine, should consent to share the lot of the poor?

"I am coming back," said Alicia, reading the distrust in Mary's eyes. "I am going away for a few hours on business, but I shall return."

"You must come back," said David, glancing up and speaking imperatively; "we can't do without you."

David meant that he could not do without her ear, her attention, her approval, her sympathy—for these are the breath of life to a poet, and this proud Alicia, who a little while ago would have withered his feeble egotism with her scorn, was now ready to give him all that he asked.

She went home to Park Lane and made a toilet that cheered the affronted spirits of her maid, who had greatly misliked her young mistress's latest freak. In fashionable luxury, and seated in the carriage, Alicia was once more a haughty young woman of the world, as wide removed as east and west from the girl in grey who sat by the doll-maker's side and learned his primitive art. It was this majestic young person who duly arrived in Paternoster Row, and, sending up her card, requested an interview with Messrs. Short and Long, the eminent publishers.

As Alicia's varied experiments in the art of rendering life worth living had not included the manufacture of a book, she scarcely knew how to approach her subject, but an air of determination seemed to fit the occasion, and she now assumed it as she swept in upon the heads of the firm in their private room.

Some correspondence had already passed between Miss Frankland and these gentlemen, and her chief anxiety now was to know how soon a certain volume of verses in which she was much interested could be issued. She found her task surprisingly easy: perhaps if she had come with a roll of manuscript in her hand she might not have had so gracious a reception; but as it was her purse that was to pay for the forthcoming edition of "Hillside Voices," Messrs. Short and Long had nothing but smiles for her. If it was her whim to play Mecænas to this young rustic—why not? Young ladies with a balance at the bankers have often spent their money more foolishly. So Alicia's fancies were all to be met and indulged; the thin river of verse was to be set off with a handsome margin, the binding to be costly and delicate. Above all, the book was promised in the shortest possible space of time.

It had need to be ready quickly if the sight of it was to cheer the poet's dying eyes, and it was this growing conviction that led Alicia, on the following day, to journey to a quiet spot near the river—a village where as a child she had spent many summer days, and where she knew of a cottage that might be hired for the remaining weeks of the autumn. She examined it carefully, and finding that it answered her expectations, she made no delay in securing it.

That done, she went back to Baron Street, and her welcome there was her best reward.

"You missed me?" she asked Mary, half-incredulously; but Mary's only answer was a smile.

Quite a week, however, elapsed before she ventured to hint at her plans, which included the kidnapping of David and the transporting of him to that cottage by the river. During that weeek she had improved her knowledge of the resources of the poor by going, under Mervyn's guidance, over St. Placide's Hospital.

The inscription that Dante placed over the door of the Inferno may be here reversed: Let him take hope with him who enters this "city of pain." The century that has witnessed this splendid outcome of Christian benevolence need surely not turn from its yet unsolved problems in despair.

Here, too, Alicia had many questions to ask and much to see that stirred the new-quickened tenderness in her heart. Mervyn looked at her with a kind of smiling reverence. The simplicity that underlay her haughtiness, the zeal with which she strove to learn and understand, moved him curiously. Yet it says something for her sound common-sense that she did not instantly resolve upon becoming a nurse—as Kitty would no doubt have done—influenced by the picturesqueness of the dress and the apparent romance of the situation. Alicia knew better the strenuous demands of that high calling, and in all humility held herself unworthy of it.

On another day she strayed into the church where Mervyn's comrade, the young priest, ministered. It was the eve of a festival, and the church was swept and garnished, and made bright with flowers. It was a glaringly hot day without, but within the big empty space it was cool, and the light came softened through coloured windows. She rested there a long time, pondering many things. Felix Mervyn gave his youth and his life to the curing of the sick bodies among the poor with whom he had cast in his lot; the young clergyman recognised nothing but the sick soul, and penance and confession were the medicines with which he sought to heal it. Perhaps he was not so mistaken as she had believed him, though she professed a wider creed. Seated here in the quiet and peace of the empty church, she recognised that a beaten man might come here and find strength to face his defeat; might even, led by ways too strange for her, see God face to face.

As she passed out, rested and soothed, she met the young priest hurrying in.

"You have never come to any of our services," he said with a little air of official reproof as he shook hands with her.

"No," said Alicia frankly, "I have not felt any impulse to come. But I will come to-night."

"Come and hear them sing," said the young man, encouraged by this promise; "they put themselves into it."

"Then it is the hymns?" said Alicia naïvely. "I had forgotten the music. Yes, I will certainly come."

Her face brightened, but his fell a little. He had an eloquent sermon in his pocket, and if his vanity was wounded for a moment that is only saying that he was human.

Alicia said to herself as she parted from him that he was very good, and if he were a little narrow, and loved millinery and form and detail—well, he was still very good.

She was by this time a perfect storehouse of information—a Parliamentary Blue Book of classified questions and answers. The housing of the poor, the schooling of the children, the people's amusements, their occupations, their opinions, their wages, their needs, their desires. The marvel was that one small head could hold it all; slowly she was assimilating, digesting her knowledge, asking herself how best to put it to practical use. She no longer asserted that she was without obligations—rather, she was making these into a law she dared not disobey.

But the first duty was towards her kindred. In a week David was so perceptibly weaker that a dawning look of terror crept into Mary's eyes, and Alicia knew that her task would be easier than she anticipated.

When she made her proposal the sister yielded at once. "For David's sake," she said; "for David's sake." She put her head down on Alicia's shoulder, and there faced the first bitterness of coming separation.

The poet was more intractable—after the manner of poets—who had the innocent belief of young authorship that his book would suffer without his supervising presence. It was only when Felix Mervyn, who was present to back Alicia with his authority, solemnly engaged to receive and post the proof-sheets with his own hands, that he gave a peevish consent.

"I suppose I must go, since Mary wishes it," he said ungraciously. "She never liked London, and yet she would come with me. She ought to have stayed at home, where she wouldn't have fretted. This air is good enough for me. London is the grandest place in the world for those who work with their brains. I never lived till I came here; but a simple country girl like Mary, how can she understand? Well, well, my dear"—he turned to her—"we'll go for a little while—to please you. Poor Mary!"

Poor David, rather! Poor poet!

(*To be continued.*)

